# Effects of Copper Metallic Nanoparticles on Structural and Optical Properties of Antimony Phosphate Glasses Co-Doped with Samarium Ions

**DOI:** 10.3390/ma13215040

**Published:** 2020-11-09

**Authors:** Petru Pascuta, Razvan Stefan, Loredana Elena Olar, Liviu Calin Bolundut, Eugen Culea

**Affiliations:** 1 Physics and Chemistry Department, Technical University of Cluj-Napoca, 103-105 Muncii Avenue, 400641 Cluj-Napoca, Romania; petru.pascuta@phys.utcluj.ro (P.P.); eugen.culea@phys.utcluj.ro (E.C.); 2 I Preclinic Department, University of Agricultural Science and Veterinary Medicine, 3-5 Calea Manastur, 400372 Cluj-Napoca, Romania; loredana.olar@usamvcluj.ro; 3 Life Sciences Institute, 3-5 Calea Manastur, 400372 Cluj-Napoca, Romania

**Keywords:** copper metallic nanoparticles, doped samarium antimony phosphate glasses, XRD, EPR, photoluminescence, UV–Vis

## Abstract

New antimony phosphate glasses doped with samarium (III) oxide and co-doped with copper metallic nanoparticles (CuNPs) were obtained by the melt quenching technique. The samples were analyzed by X-ray diffraction analysis (XRD), electron paramagnetic resonance (EPR), ultraviolet-visible (UV–Vis) and photoluminescence (PL) spectroscopies. XRD data suggested that all the obtained samples showed an amorphous nature. EPR data suggested the existence of Cu^2+^ ions octahedrally surrounded by six oxygen atoms. The dipole–dipole interactions between Cu^2+^ ions were predominant. UV–Vis spectra revealed the presence of Sm^3+^ and Cu^2+^ ions in the samples. The values for nephelauxetic and bonding parameters were also calculated. The negative values obtained for bonding parameter indicate an ionic character of the bonds from the glass network. Photoluminescence spectra exhibited emissions from samarium ions and revealed the influence of dopant nature on of rare-earth ions emissions. The obtained results indicate that the studied materials are suitable for solid state lasers.

## 1. Introduction

At the present time, investigations of amorphous and crystalline materials are of great importance. The antimony phosphate glasses doped with rare-earth (RE) ions and co-doped with some metallic nanoparticles are interesting materials. Glasses containing antimony oxide present multiple applications, especially in photonics [1,2]. Antimony is a metal from the p block of elements that has free p orbitals with multiple possibilities for formation of chemical bonds, presenting two oxidation states: III and V. Antimony (III) oxide, Sb_2_O_3_, is considered a good glass former oxide [3,4]. The glasses based on antimony (III) oxide have low phonon energy, good thermal stability, and high linear and non-linear refractive indexes [5,6]. In addition, such glasses show potential in the field of optical amplification, being suitable for telecommunication devices [7]. Sb_2_O_3_ seems to be a good reducing agent, being useful in the preparation of glasses containing metallic nanoparticles [4,8,9].

Among RE ions used as dopants for glasses with optical applications, Sm^3+^ occupies an important place due to its energy levels that determine fluorescence properties (strong yellow and red emissions). The glasses doped with Sm^3+^ ions can be useful for fiber lasers and planner waveguides [10,11]. Due to the 4f electronic configuration, Sm^3+^ exhibits characteristic and intense absorption bands in the UV to blue region [12].

In recent years, research has been conducted on embedding metallic nanoparticles (NPs) in the glass network. In this way, glasses doped with RE and co-doped with NPs became attractive due to the enhancement effect that seems to have the NPs on the luminescence emissions of the RE ions [13,14,15]. The NPs’ co-doping of RE-containing glasses increased the domains of application for this kind of material: three-dimensional multicolored industrial art objects, photochromatic materials, and the color glass recycling industry [16,17,18]. 

The aim of this work was to prepare and study new antimony phosphate glasses doped with samarium (III) oxide and co-doped with copper metallic nanoparticles. The mentioned glasses were investigated by X-ray diffraction (XRD), UV–Vis, luminescence (PL), and electron paramagnetic resonance (EPR) measurements. The obtained data were used to determine the influence of the increasing content of the copper nanoparticles on the optical and structural properties of the obtained glasses.

## 2. Materials and Methods

Samples of the (CuNPs)_x_·(P_2_O_5_)_35_·(Sb_2_O_3_)_(64−x)_·(Sm_2_O_3_)_1_ system (code: CuNPsPSbSm) with 0≤ x ≤15 mol % were obtained using the melt quenching technique. Chemical reagents used (Sm_2_O_3_, Sb_2_O_3_, P_2_O_5_, and copper nanoparticles (CuNPs) (particle size of 20–50 nm)) were high-purity Alfa Aesar (Thermo Fisher GmBH, Kandel, Germany) products. The oxides were mixed in stoichiometric amounts using an agate mill (Lemke GmBH, Bruchweiler, Germany) to obtain homogenous mixtures. These mixtures were melted in corundum crucibles at 900 °C for 15 min using an electric furnace. Then, the melts were cooled out at room temperature by being poured onto a stainless-steel plate.

All the samples were analyzed by XRD, EPR, UV–Vis and luminescence spectroscopies. A Shimadzu 6000 XRD diffractometer (Shimadzu Corporation, Kyoto, Japan) was used to investigate the nature of samples and to determine the occurring crystalline phases. EPR measurements were performed using powder samples and were carried out in the X-band (~9.79 GHz) at room temperature using a Bruker E-500 ELEXSYS spectrometer (Bruker UK Limited, Coventry, UK). The spectra processing was performed by the Bruker Xepr software (Bruker UK Limited, Coventry, UK). UV-VIS absorption spectra were obtained using a Jasco V-550 spectrometer (Jasco Europe s.r.l., Cremella, Italy) in the 300–1600 nm wavelength range with a resolution of 2 nm. The photoluminescent behavior of samples was studied using an Able Jasco FP 6500 spectrofluorometer (Jasco Europe s.r.l., Cremella, Italy) with a Xe lamp. Other details for each investigation method and apparatus used can be found in our team’s earlier publications [19,20,21,22].

## 3. Results and Discussion

### 3.1. XRD Data

**Figure 1:**
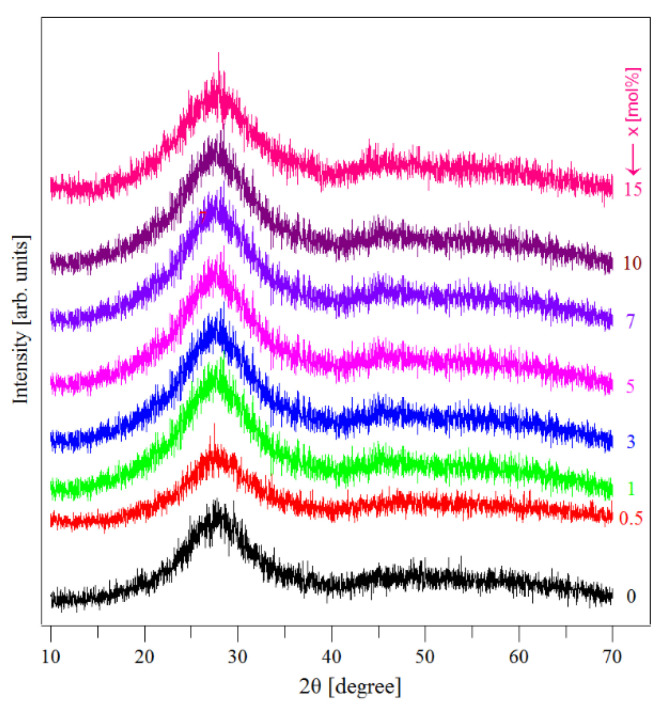
XRD patterns of the (CuNPs)_x_·(P_2_O_5_)_35_·(Sb_2_O_3_)_(64−x)_·(Sm_2_O_3_)_1_ glasses.

Figure 1 presents the XRD spectra of the as-synthesized samples. The X-ray diffraction spectra exhibited a broad hump-like feature, revealing the amorphous nature of the studied samples. The XRD diffractograms not indicating the presence of the CuNPs was somewhat surprising. However, an explanation is offered below. 

### 3.2. EPR Data

**Figure 2:**
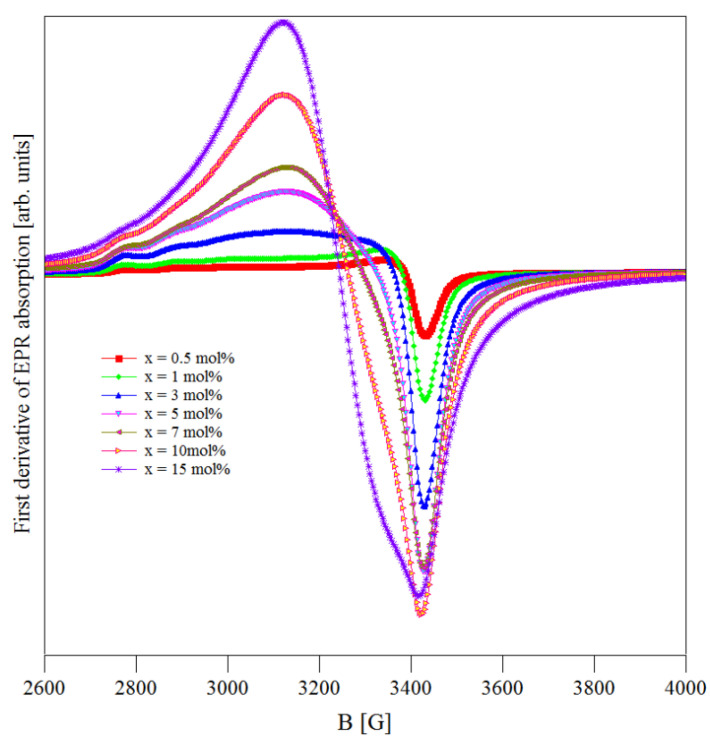
EPR absorption spectra of Cu^2+^ ions in the (CuNPs)_x_·(P_2_O_5_)_35_·(Sb_2_O_3_)_(64−x)_·(Sm_2_O_3_)_1_ glasses.

The EPR spectra of (CuNPs)_x_·(P_2_O_5_)_35_·(Sb_2_O_3_)_(64−x)_·(Sm_2_O_3_)_1_ glasses recorded at room temperature are shown in Figure 2. These spectra were formed due to copper ions present in the samples in their 2+ valence state. This assumption is well supported by their close resemblance with the spectra reported for some copper boro-tellurite [23] and copper tellurite [24] glasses containing Cu^2+^ ions. No signals related to the presence of some EPR active antimony ions were noticed [25,26]. 

The appearance of Cu^2+^ ions in the (CuNPs)_x_·(P_2_O_5_)_35_·(Sb_2_O_3_)_(64−x)_·(Sm_2_O_3_)_1_ glasses was due to the oxidation process that affected the CuNPs from these samples. It is known that common metal nanoparticles, including CuNPs, are liable to the oxidation process that starts immediately after their preparation. Thus, at the surface of the CuNPs, a thin layer of Cu_2_O and CuO will appear, meaning that, besides the Cu^0^ species, Cu^+^ and Cu^2+^ will be present. Under appropriate conditions, the oxidation process may continue during the melting process and this explains the increase in the amount of Cu^+^ and Cu^2+^ ions present in the samples. This sensitivity of copper to the oxidation process is explained by the existence of several reports concerning the presence of copper ions in multiple valence states (Cu^0^, Cu^+^ and Cu^2+^) in oxide glasses [27,28,29,30].

In general, the Cu^2+^ ions from oxide glasses are located at sites with an octahedral or a tetrahedral symmetry and an axial distortion. Consequently, their EPR spectra are analyzed using an axial spin Hamiltonian. Due to a hyperfine coupling between the electronic spin S = 1/2 and the nuclear spin I = 3/2 of the Cu^2+^ ions, their EPR spectrum showed a hyperfine structure consisting of four parallel and four perpendicular components [27,28,29,30].

The EPR spectra recorded for the (CuNPs)_x_·(P_2_O_5_)_35_·(Sb_2_O_3_)_(64−x)_·(Sm_2_O_3_)_1_ glasses (Figure 2) represent a superposition of two contributions. First, there was an EPR absorption with a poorly resolved hyperfine structure (hfs) located at *g* ≈ 2 due to the isolated Cu^2+^ species [27,28,29,30]. Second, there was a broad and symmetric EPR absorption line located at *g* ≈ 2.1 with an isotropic *g* due to the coupled/clustered copper ions [23,24,27,28,29,30,31]. The main contribution was from the coupled/clustered ions, suggesting that their number was higher than that of the isolated ions.

As mentioned, the first component of the EPR spectra, due to the isolated Cu^2+^ ions, was asymmetric and showed a partially resolved hfs with two *g* (*g*_||_ and *g*_⊥_ for the isolated Cu^2+^ ions and g for the coupled/clustered species). This signal is characteristic of Cu^2+^ ions situated in sites with an axially distorted octahedral symmetry [31,32,33,34].

**Figure 3:**
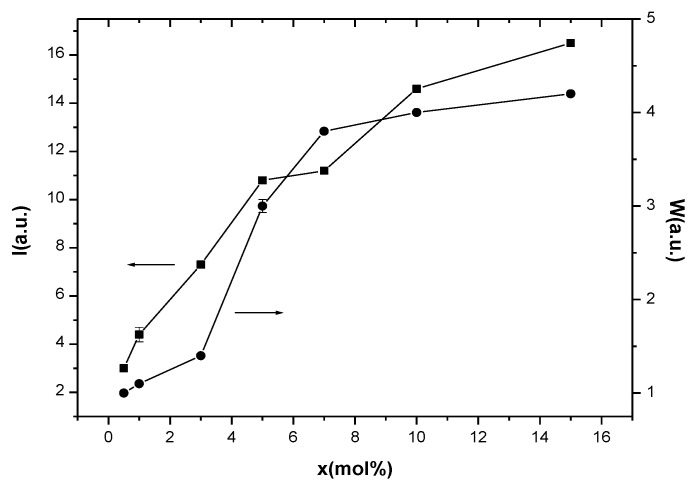
Composition dependence of intensity and line-width of EPR absorption of (CuNPs)_x_·(P_2_O_5_)_35_·(Sb_2_O_3_)_(64−x)_·(Sm_2_O_3_)_1_ glasses. The lines are drawn as a visual guide.

The processing of the EPR spectra and the calculation of the important EPR parameters were realized using Bruker Xepr software and considering an axial Hamiltonian and the resonance formula (h*ν* = gβB, where g = *g* factor, β = Bohr magneton, h = Planck constant, *ν* = frequency, and B = resonance field). Following this procedure, we obtained the values for the *g* factors (*g*_||_ and *g*_⊥_ for the isolated Cu^2+^ ions and *g* for the coupled/clustered species), which are presented in Table 1, and the values for the integral intensities (I) and the peak-to-peak line widths (W), which are presented in Figure 3.

As mentioned, to obtain more information concerning the location of the Cu^2+^ ions in the (CuNPs)_x_·(P_2_O_3_)_35_·(Sb_2_O_3_)_(64−x)_·(Sm_2_O_3_)_1_ glasses, we calculated the *g*_||_ and *g*_⊥_ factor values, which are presented in Table 1.

These data revealed the *g*_||_ > *g*_⊥_ > *g_e_* relation (where *g_e_* = 2.0023 represents the *g* factor of free electron), suggesting an axially distorted octahedral symmetry of the Cu^2+^ ions’ microvicinities [27,29]. The compositional variation of the EPR signals’ intensities (I) and line widths (W) of the EPR absorptions for the studied samples is shown in Figure 3.

It is known that the EPR absorption intensity, I, is proportional to the amount of paramagnetic ions that generate the resonance absorption. In our case, I increases with increasing samples’ CuNPs content, suggesting the increase of the number of Cu^2+^ ions in the samples. The analysis of the compositional evolution of the EPR linewidth, W, correlated with the EPR signal intensity may provide important information concerning the nature of the interactions between the paramagnetic ions, dipole-dipole, or exchange types. The compositional evolution of I and W shown in Figure 3, where both I and W increase with increased CuNPs and, consequently, the Cu^2+^ content of samples, suggests that the dipole-dipole type interactions between the Cu^2+^ ions are predominant [28,29]. Note also that the presence of Cu^+^ ions may influence the EPR line width. Although the Cu^+^ ions are EPR-silent, these ions interact with the Cu^2+^ ones and the Cu^+^–Cu^2+^ interactions will produce a supplementary broadening of the EPR absorption [31,33].

### 3.3. UV–Vis Data

**Figure 4:**
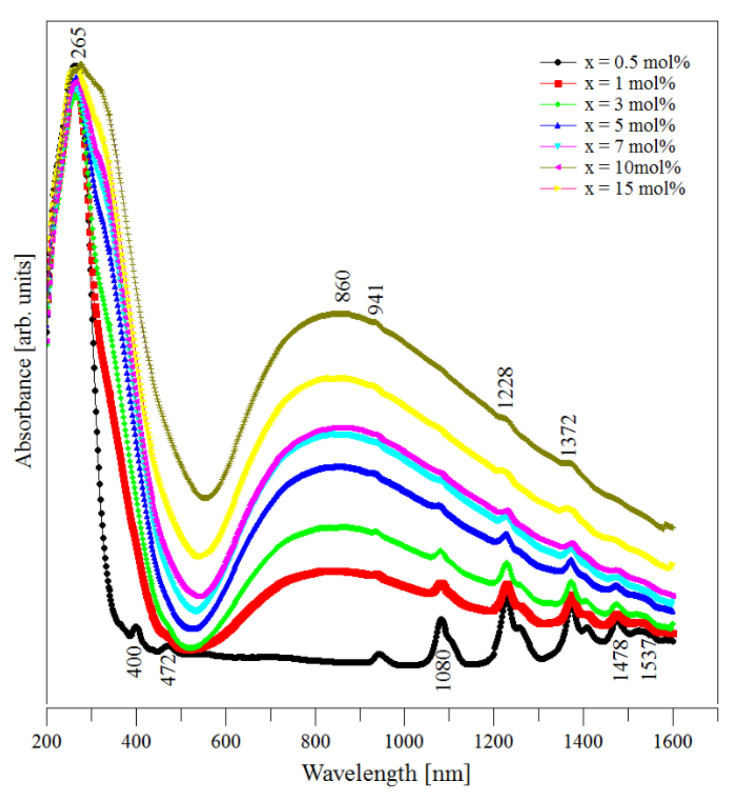
UV–Vis absorption spectra of the (CuNPs)_x_·(P_2_O_5_)_35_·(Sb_2_O_3_)_(64−x)_·(Sm_2_O_3_)_1_ glasses.

The UV–Vis spectra of (CuNPs)_x_·(P_2_O_5_)_35_·(Sb_2_O_3_)_(64−x)_·(Sm_2_O_3_)_1_ glasses are presented in Figure 4. These spectra were recorded at room temperature.

The spectrum of the sample without copper metallic nanoparticles (CuNPs) presented nine important peaks that corresponded to some f–f electronic transitions from the ^6^H_5/2_ ground state to different excited electronic levels of the Sm^3+^ ions. These absorption bands are situated mainly in the visible and near infrared regions. According to the literature [35,36,37], we assigned the mentioned absorption bands as follows: 265 nm (^6^H_5/2_ → ^2^H_9/2,_^4^I_9/2_), 400 nm (^6^H_5/2_ → ^6^P_3/2_), 472 nm (^6^H_5/2_ → ^4^I_11/2_), 941 nm (^6^H_5/2_ → ^6^F_11/2_), 1080 nm (^6^H_5/2_ → ^6^F_9/2_), 1228 nm (^6^H_5/2_ → ^6^F_7/2_), 1372 nm (^6^H_5/2_ → ^6^F_5/2_), 1478 nm (^6^H_5/2_ → ^6^F_3/2_), and 1537 nm (^6^H_5/2_ → ^6^H1_5/2_). In the case of samples doped with CuNPs, fewer absorption peaks were observed due to the overlaps with the peaks from the Cu^2+^ ions embedded in the glass interstices.

The predominant absorption was located around 860 nm and corresponds to the ^2^E_g_ → ^2^T_2g_ electronic transition belonging to Cu^2+^ ions. This indicated that, in our samples, the Cu^2+^ ions occupied sites that are octahedrally surrounded by six oxygens and that all the copper-oxygen bonds are equals. By increasing the samples’ level of CuNPs content, the position of this absorption band remained unchanged while its intensity increased. The compositional evolution of this absorption band was due to the increase in the Cu^2+^ ion concentration in the samples. This behavior is due to a part of Cu^0^ from the nanoparticles being oxidized from Cu^0^ to Cu^2+^ at the melting temperature, as was previously recorded for other oxide glasses (i.e., copper phosphate glasses [38] and copper aluminosilicate glasses [39]). Note that the literature data [4,8,9] mention that Sb_2_O_3_ is a very good reducing agent. Under such conditions, copper should be present as Cu^0^ due to the antimony (III) oxide content of samples. However, in our case, the thermodynamic factor seems to favor the oxidation process of Cu^0^ to Cu^2+^. Another possible explanation for the compositional evolution of the intensity of the absorption band from 860 nm can be related to the antimony content of samples decreasing by increasing their CuNPs content. Thus, the concentration of the reducing agent (antimony) decreases and its reducing capacity becomes weaker in the samples with higher CuNPs contents.

For a better understanding of the structure of the studied glasses, the nephelauxetic (*β*) and bonding parameters (*δ*) were calculated for all the prepared samples according to the method presented in the literature [20,36,40]. Thus, to calculate the nephelauxetic ratio of the samples the $\beta =\frac{\nu_{c}}{\nu_{a}}$ relation was used [40], where $\nu_{c}$ is the wavenumber (in cm^−1^) of a particular transition from the UV–Vis spectrum of the studied sample and $\nu_{a}$ is the wavenumber for the corresponding transition in an aquatic environment [36]. Using the average values of *β* ($\bar{\beta }$), the bonding parameter (δ) was calculated using the formula [40]:

$$\delta =\;\frac{1-\bar{\beta }}{\bar{\beta }}\;\times \;100$$

Note that the *δ* values suggest the ionic or covalent character of the chemical bonds in the samples: ionic for negative δ values and covalent for the positive ones. In our case, the calculated bonding parameter values were negative, suggesting that the glass network presented an ionic bond characteristic. These values increased by increasing the CuNPs content of samples from *δ* = −1.05974 for the CuNPs free sample up to *δ* = −1.5192 for the sample with 15 mol % CuNPs, suggesting an increase in the ionic bond characteristic for higher CuNPs contents. This behavior was expected since, by increasing the CuNPs content of samples, the copper replaces the antimony and the metallic character of copper is higher than that of antimony.

Note that both the UV–Vis and EPR data suggest an intense oxidation process that affects the CuNPs. This finding may explain the lack of crystalline features characteristic of CuNPs in the XRD diffractograms of the studied samples. To explain this finding, we assumed that the oxidation of CuNPs is so intense that it decreases the amount of the CuNPs in the samples at a level that is lower than the detection limit of XRD (estimated about 5%) despite the short melting time (15 min). This is an important observation considering the technological interest that exists in replacing the use of noble metal (Au, Ag) nanoparticles with cheaper common metal nanoparticles. Thus, the mentioned replacement may be operated keeping in mind the important effects of potential oxidation processes that will modify the amount of common metal nanoparticles.

### 3.4. Luminescence Data

**Figure 5:**
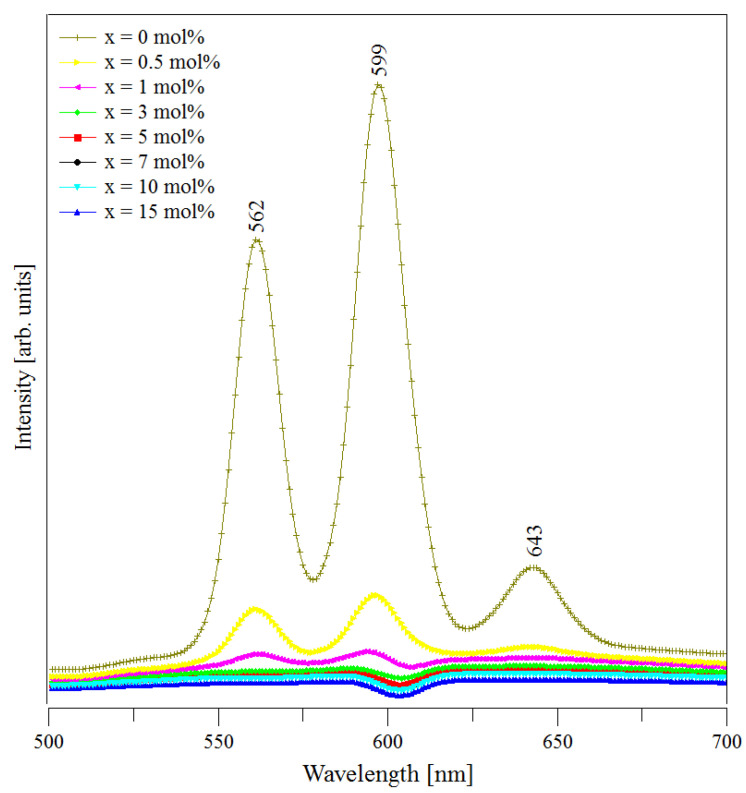
Photoluminescence emissions of the (CuNPs)_x_·(P_2_O_5_)_35_·(Sb_2_O_3_)_(64−x)_·(Sm_2_O_3_)_1_ glasses.

The luminescence spectra of the (CuNPs)_x_·(P_2_O_5_)_35_·(Sb_2_O_3_)_(64−x)_·(Sm_2_O_3_)_1_ glasses are presented in Figure 5. These spectra were recorded at room temperature using an excitation wavelength of 403 nm, which may produce the excitation up to the ^4^K_11/2_ level. The observed emissions occurred only from the ^4^G_5/2_ energy level, as was previously reported for some other glasses (e.g., lithium borate glasses doped with gadolinium and samarium ions [41]). This phenomenon is possible due to some non-radiative processes that occur from the ^4^K_11/2_ to the ^4^G_5/2_ energy level.

The obtained spectra showed the emission peaks of all three samples. These peaks occur due to some transitions from the ^4^G_5/2_ excited level to different energy levels of the Sm^3+^ ions. According to the literature data [20,35,37,41], they are assigned as follows:

- 562 nm assigned to the ^4^G_5/2_ → to the ^6^H_5/2_ ground state transition; it is called also the zero-zero band and represents a forbidden transition;
- 599 nm assigned to the ^4^G_5/2_ → to the ^6^H_7/2_ (excited level) transition, a magnetic dipole transition;
- 643 nm assigned to the ^4^G_5/2_ → to the ^6^H_9/2_ (excited level) transition, an electric dipole transition.

**Figure 6:**
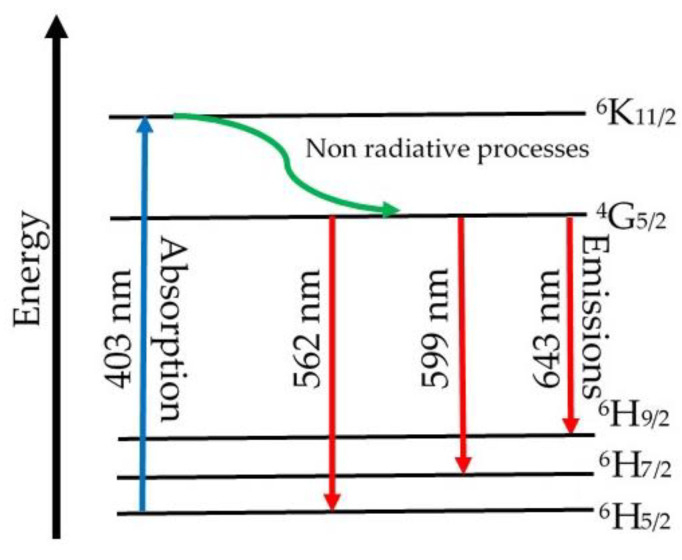
Energy level diagram with absorption and emissions for Sm^3+^ ions in (CuNPs)_x_·(P_2_O_5_)_35_·(Sb_2_O_3_)_(64−x)_·(Sm_2_O_3_)_1_ glasses.

Once the metallic copper nanoparticles were added to the batch, the intensity of the emission peaks decreased. The increase of samples’ CuNPs content led to the further decrease in intensity of the Sm^3+^ ion emissions. The quenching effect was due to non-radiative relaxation processes consisting of (i) multiphonon relaxations and (ii) energy transfer between the Sm^3+^ ion pairs via cross-relaxation processes [42]. Since the energy gap of Sm^3+^ ions (the difference between the fluorescent ^4^G_5/2_ level and the next lower ^6^F_1/2_ level is 7300 cm^−1^) was several times higher than the highest phonon energy level in phosphate glasses (1300 cm^−1^) [43] and stibium glasses [44], multiphonon relaxation may be considered negligible. Thus, in our case, the energy transfer through cross-relaxation may be considered responsible for the observed quenching effect. Figure 6 presents a possible diagram of the energy level, including absorption and emissions for Sm^3+^ ions in (CuNPs)_x_·(P_2_O_5_)_35_·(Sb_2_O_3_)_(64−x)_·(Sm_2_O_3_)_1_ glasses. However, to make a rigorous assumption concerning the potential cross-relaxation channels, a more detailed study of the absorption, excitation, and emission behaviour of Sm^3+^ ions in the studied glasses is necessary. Since the quenching occurred by increasing the samples’ CuNPs content, we assumed that the higher amount of copper nanoparticles “shortens” the distances between the Sm^3+^ ions from the glass network, reducing the non-radiative processes and, as a consequence, the intensity of emission peaks.

Note that a second mechanism may also be considered to explain the decrease in the intensity of emission peaks with increasing CuNPs content in samples. Thus, during the melting of samples, the CuNPs were oxidized to Cu^2+^ ions due to the thermodynamic factor that favors the oxidation processes (as previously reported by the authors) [39]. Note that the presence of a considerable amount of Cu^2+^ ions in our samples was ascertained by the EPR and UV–Vis data. In this case, we did not observe a quenching effect; instead, we observed a decrease in the emission intensities due to the decrease of the amount of CuNPs.

The most intense emission from our spectra was the peak from 599 nm in all the cases. The second band in intensity was the peak from 562 nm. In our study, the Sm^3+^ ions presented orange and green luminescent transitions situated in the visible region of the spectrum. Therefore, this kind of material is suitable for solid state lasers.

## 4. Conclusions

New stibium-phosphate glasses doped with samarium (III) ions and co-doped with CuNPs were prepared by using the melt-quenching technique. Their structure and optical properties were studied by XRD, EPR, UV–Vis, and luminescence measurements. XRD patterns revealed the amorphous character of all the studied samples. EPR data confirmed the presence of Cu^2+^ ions situated in octahedral sites in the samples and suggested that the dipole–dipole type interactions between the Cu^2+^ ions were predominant. UV-vis spectra exhibited some f-f electronic transitions assigned to the Sm^3+^ ions. A part of them overlapped the peak due to the Cu^2+^ ions that indicated the presence of the Cu^2+^ ions located at sites that are octahedrally surrounded by six oxygens. The calculated bonding parameter had a negative value indicating that the bonds from the glass network had an ionic character. The photoluminescence spectra showed that the most intense emissions are the orange and green ones from the visible region of the spectra, making these materials suitable for solid state lasers. 

## Figures and Tables

**Table 1:** The *g*, *g*_||_ and *g*_⊥_ factors values for the CuNPsPSbSm glasses.

x (mol %)	g_||_	g_⊥_	g
0.5	2.398	2.083	
1	2.404	2.087	
3	2.407	2.097	
5	2.410	2.104	
7			2.145
10			2.161
15			2.174
